# Identification of pre-fertilization reproductive barriers and the underlying cytological mechanism in crosses among three petal-types of *Jasminum sambac* and their relevance to phylogenetic relationships

**DOI:** 10.1371/journal.pone.0176026

**Published:** 2017-04-18

**Authors:** Yanming Deng, Xiaobo Sun, Chunsun Gu, Xinping Jia, Lijian Liang, Jiale Su

**Affiliations:** 1 Provincial Key Laboratory for Horticultural Crop Genetic Improvement, Institute of Leisure Agriculture, Jiangsu Academy of Agricultural Sciences, Nanjing, Jiangsu, China; 2 Institute of Botany, Jiangsu Province and Chinese Academy of Sciences, Nanjing, Jiangsu, China; United States Department of Agriculture, UNITED STATES

## Abstract

Crosses among single-, double- and multi-petal jasmine cultivars (*Jasminum sambac* Aiton) are unable to easily generate hybrids. To identify the reproductive barriers restricting hybrid set, dynamic changes in jasmine pollen viability and pistil receptivity were compared at different flowering stages. Pollen-pistil interactions in six reciprocal crosses were also investigated to characterize pollen-stigma compatibility. Additionally, paraffin sections of pollinated embryo sacs were prepared for subsequent analyses of developmental status. Furthermore, pistil cell ultrastructural characteristics were observed to reveal cytological mechanism regulating pistil receptivity and the pollen-pistil interactions. We observed that pollen viability and stigma receptivity varied depending on petal phenotype and flowering stage and were easily lost during flowering. Different reciprocal crosses exhibited varied pollen-stigma compatibilities according to the pollen germination rates. Although some pollen grains germinated normally on maternal stigmas, the pollen tubes were arrested in the pistils and were unable to reach the ovaries. Additionally, the embryo sacs remained unfertilized until degenerating. Therefore, jasmine crosses are affected by pre-fertilization reproductive barriers. Low pollen fertility and poor stigma receptivity are detrimental to pollen germination and pollen-pistil compatibility, indicating they are two factors affecting hybrid set. Ultrastructural observation of the pistil cells revealed that cell death occurred during flowering. Thus, the early and rapid senescence of pistils is likely responsible for the decreased pistil receptivity and inhibited pollen tube growth. These findings may be relevant for future jasmine hybridizations. They provide new insights for the development of methods to overcome reproductive barriers and may also be useful for clarifying the phylogenetic relationships among jasmine cultivars with differing petal phenotypes.

## Introduction

Jasmine (*Jasminum sambac* Aiton, Oleaceae) is an important commercial ornament plant that has been used in floral bouquets in many countries. In China, jasmine plants exhibit single-petal (SP), double-petal (DP), and multi-petal (MP) phenotypes [1]. Each jasmine phenotype is associated with specific characteristics related to growth, anthesis, stress tolerance, ecological adaptation and economic value [1–5]. For example, the DP jasmine plants produce the most flowers, and are the most tolerant to abiotic stresses such as low temperatures, water deficiency and limited irradiance. The SP flower is considered to be the most fragrant, while the MP jasmine plants have the highest ornamental value because of their supernumerary petals [5–7].

There has recently been a demand for novel jasmine cultivars that combine the best attributes of each of the jasmine phenotypes, including the resistance of DP plants, the beauty of MP flowers, and the fragrance of SP flowers. Sexual hybridization is well known as one of the most efficient methods for breeding horticultural cultivars, which suggests it offers the best chance of creating novel jasmine cultivars using the currently available jasmine lines with differing petal phenotypes. However, previous hybridization attempts have failed, with no hybrids being produced (unpublished data). Because reproductive barriers considerably decrease the efficiency of cross breeding, it is possible that these barriers affect jasmine crosses to prevent the generation of hybrids. Therefore, characterizing the nature of these particular barriers will be important for developing effective mitigating strategies.

Pollen viability is an important factor closely related to reproductive barriers during crosses, especially pre-fertilization barriers [8–9]. Low pollen viability decreases the probability of pollen germination on a stigma [9–11]. Previous studies have revealed that the pollen grains of DP jasmine plants exhibit low fertility levels, which is detrimental for seed sets [12–14]. An analysis of the anatomy of DP jasmine plants revealed that some tetrads form a pollen massula instead of free microspores, and some microspores developed abnormally, which together reduces pollen fertility [14]. However, pollen viability is variable during flowering [9,10,15]. Therefore, determining the dynamic changes in pollen viability during the flowering stage is necessary to comprehensively characterize reproductive barriers, and may also be helpful for selecting fertile pollen grains.

Stigma receptivity and pollen-stigma interactions (including the number of germinated pollen grains, pollen tube growth, and the callose reaction) are important factors considerably affecting hybridizations, and are frequently associated with pre-fertilization barriers [8,9]. Stigma receptivity is crucial for the capture and normal germination of pollen grains, which indicates its importance for pollination [8,9,16,17]. The pollen-stigma interaction is an obvious and useful indicator for reproductive barriers because of its role during pollen germination and pollen tube growth. Analyses of pollen-stigma interactions have helped to characterize the pre-fertilization barriers that decrease seed production in many plant species [18–20]. For example, a low rate of normal pollen germination, the development of coiled and twisted pollen tubes, and the deposition of callose on stigmatic surfaces are potential markers for the incompatibility between species or cultivars [9,19–22]. Therefore, characterizing the stigma receptivity and pollen-stigma interactions of jasmine plants with differing petal phenotypes is essential for revealing the reproductive barriers to hybridizations.

The ultrastructural characteristics of pistil cells influence stigma receptivity and pollen-stigma interactions [23]. Considering only one pistil and numerous pollen grains are developed simultaneously in one flower, stigma receptivity may have a prominent role during successful artificial hybridizations. Therefore, observing the dynamic ultrastructural changes in pistil cells may help to elucidate the cytological mechanism underlying stigma receptivity and pollen-stigma interactions. It may also be useful for selecting suitable pistils for pollinations to overcome hybridization barriers.

In this study, we assessed pollen viability and stigma receptivity during flowering, pollen-stigma interactions, and the developmental status of female gametophytes following pollination, as determinants for the pre-fertilization barriers. Based on these analyses, we clarified the cytological mechanism regulating decreases in pistil receptivity. More importantly, we confirmed the hypothesis that dynamic ultrastructural pistil cell characteristics can adversely affect pistil receptivity and pollen-pistil interactions, resulting in a lack of hybrids from jasmine crosses. Our findings may be relevant to the development of effective methods to overcome reproductive barriers during the hybridization of *J*. *sambac* cultivars. They may also be useful for determining the phylogenetic relationships among the three jasmine petal-types.

## Materials and methods

### Plant materials

**Fig 1:**
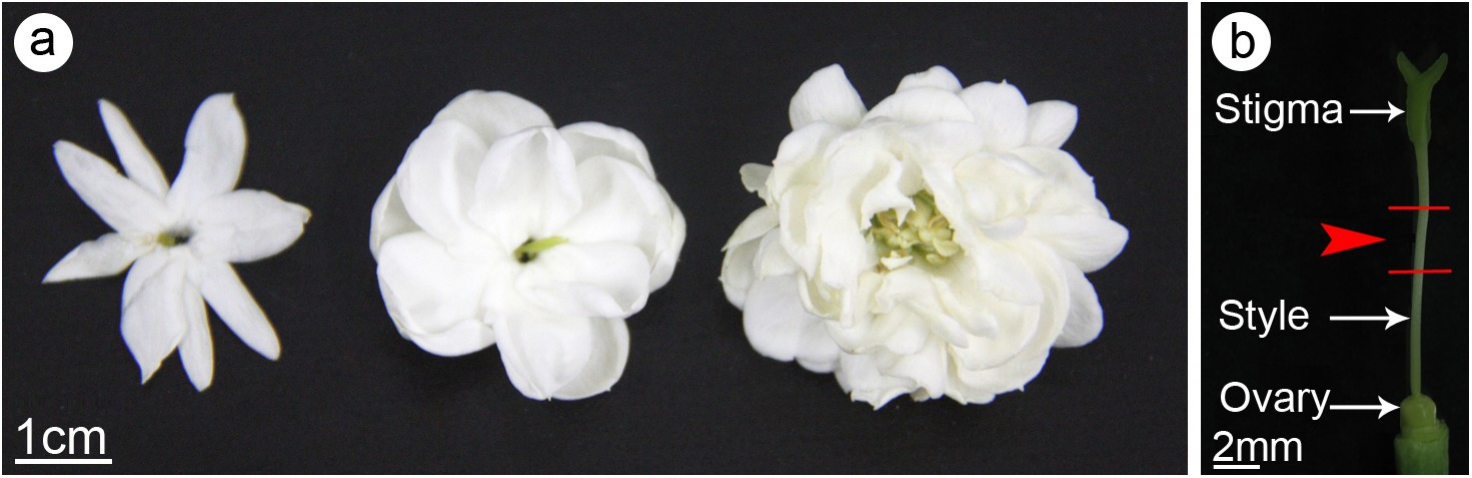
The flowers of jasmine cultivars exhibit differing petal phenotypes. (**a**) The single-petal (SP, *left*), double-petal (DP, *middle*) and multi-petal (MP, *right*) flowers. (**b**) A pistil of DP flower showing the sampled position (indicated by *arrow head*) of the style for ultrastructural observation.

The jasmine cultivars named ‘Danbanmoli’, ‘Shuangbanmoli’ and ‘Duobanmoli’ (Fig 1A) were cultivated and preserved at the Preservation Centre of the Jasmine Germplasm Resource at the Jiangsu Academy of Agricultural Sciences in Nanjing, China (latitude: 32°05′N, longitude: 118°08′E; 68 m above sea level). ‘Shuangbanmoli’ is the most popular and representative cultivar among DP jasmine plants, while ‘Danbanmoli’ and ‘Duobanmoli’ are respectively the only cultivars exhibiting the SP and MP phenotypes [5]. The jasmine plants were grown and managed as previously described [6–7]. The plants used in the present study were produced from cuttings taken from 3-year-old plants that exhibited the potential to produce high flower yields. We cultivated 100 vigorous plants from the same clone of each cultivar in 20 plastic pots (height: 0.15 m, diameter: 0.17 m), and collected representative flowers from multiple plants. Pre-tests on floral characteristics indicated there were no significant differences within each genetic group.

### Determination of pollen viability and pistil receptivity

We used several methods to estimate jasmine pollen viability in preliminary tests. These methods included the use of triphenyl tetrazolium chloride, the peroxidase reaction, and germination in different media [13]. We determined that germinating in BK medium (containing 100 mg l^-1^ H_3_BO_3_, 100 mg l^-1^ KNO_3_, 200 mg l^-1^ MgSO_4_ 7H_2_O and 300 mg l^-1^ Ca(NO_3_)_2_ 4H_2_O) was the most suitable method for assessing pollen viability. This method was used for the duration of this study. Fresh pollen grains were collected from flowers during the following four stages: *Stage 1*, one day before flowers partially opened; *Stage 2*, partially opened flowers; *Stage 3*, fully opened flowers; and *Stage 4*, one day after flowers fully opened. During each stage, pollen grains were collected at 08:00–10:00 with a soft brush. The pollen grains were then germinated on glass slides, and subsequently incubated at 30°C for 4 h. Pollen grains that produced a tube longer than its diameter were considered fertile. The fertile pollen grains in 10 optical fields were counted using a BX43 microscope (Olympus, Tokyo, Japan). Each optical field consisted of at least 50 pollen grains. The germination rate was calculated by dividing the number of germinated pollen grains by the total number of pollen grains.

Pistil receptivity was analyzed using manual pollinations with fresh samples of the most fertile pollen grains. The pistils were pollinated at 08:00–10:00 during the same four stages described earlier. We sampled the pollinated pistils 8 h later. The pistils were immediately fixed in FAA solution (formalin/alcohol/glacial acetic acid = 90/5/5, v/v/v) and observed using an Axioskop 40 fluorescence microscope (Carl Zeiss, Jena, Germany). An aniline blue staining method was used to count the germinated pollen grains [9]. Pistil receptivity was based on the ratio of the number of germinated pollen grains to the total number of pollinated pollen grains. Ten pistils were tested at each stage and the experiment was repeated three times.

### Artificial crosses

Artificial reciprocal crosses were completed for the following six combinations: ‘Danbanmoli’ and ‘Shuangbanmoli’ [hereafter referred to as “SP × DP” (pollen receptor × pollen donor) and “DP × SP”), ‘Danbanmoli’ and ‘Duobanmoli’ (“SP × MP” and “MP × SP”), and ‘Shuangbanmoli’ and ‘Duobanmoli’ (“DP × MP” and “MP × DP”). A total of 120 flowers of each maternal parent were emasculated and covered with paper bags during the stage before anthesis, but after the anthers were removed. Pistils were artificially pollinated using fresh pollen grains collected from paternal flowers, which were also covered with paper bags before anthesis. The pollinated flowers were subsequently bagged.

### Observation of the pollen-pistil interaction

To observe pollen-pistil interactions in the various crosses, pollen grains with the highest germinability were collected from flowers and used to artificially pollinate the most receptive stigmas. Pollen germination on stigmas was investigated using a slightly modified published procedure [20]. Samples were collected at 1, 2, 4, 8, 12 and 24 h after pollination (HAP). Each sample included 20 pistils, of which 10 were immediately fixed in FAA solution and observed using an Axioskop 40 fluorescence microscope. The pollen grains and tubes were analyzed according to the aniline blue staining method. The other 10 pistils were immediately fixed in 2.5% glutaraldehyde (0.1 M phosphate buffer, pH 7.2) and analyzed by an S-3000N scanning electron microscope (SEM) (Hitachi, Tokyo, Japan). The fixed SEM samples were dehydrated in an ethanol series (40, 70, 90 and 100%) for 15 min at each concentration, and then subjected to critical point drying before being coated with gold and analyzed using SEM [20]. The same number of self-pollinated pistils (i.e., SP × SP, DP × DP, and MP × MP) was treated as described above as control samples. Each experiment was repeated three times.

### Observation of pistil cell ultrastructures

Pistils of each cultivar were collected during the same four stages described earlier, and analyzed by transmission electron microscope (TEM) as previously described [6]. The samples were collected from two parts of each pistil [i.e., one randomly selected stigma and the style segment (2–3 mm long) located 2–3 mm from the stigma] (Fig 1B). The samples were immediately immersed in 2.5% (v/v) glutaraldehyde (in 0.1 mol L^-1^ phosphate buffer, pH 7.2) for 24 h, and then stored at 4°C until used. The samples were washed with 0.1 M phosphate buffer (pH 7.2) and then post-fixed in 1.5% osmium tetroxide for 4 h. They were subsequently treated with a graded series of PHEM buffer (60 mM PIPES, 25 mM HEPES, 10 mM EGTA and 2 mM MgCl_2_, pH 7.0) and ethanol solutions, and then embedded in Epon 812 resin. Cross sections were cut to a thickness of 80 nm and stained with uranyl acetate and lead citrate. The sections were then analyzed and imaged using an H7650 TEM (Hitachi, Tokyo, Japan).

### Anatomical observation of ovules

To determine whether fertilization occurred, we examined the developmental status of female gametophytes. For each cross combination, we prepared paraffin sections of 100 plump ovaries harvested at 3, 4, 5, and 6 days after pollination (DAP). Samples were treated as described by Deng et al. [20]. Longitudinal sections of ovules were cut to a thickness of 10 μm using a KD-2258 rotary microtome (Kedi, Zhejiang, China), stained with Heidenhain’s hematoxylin solution, and observed using a BX43 optical microscope.

### Data analysis

Data were analyzed by a two-way analysis of variance (ANOVA) using SPSS 17.0 (SPSS Inc., Chicago, IL, USA) for Windows. Duncan’s multiple range test was adopted to determine significant differences between means (*P* < 0.05 or 0.01). The data are presented as the mean value ± standard deviation.

## Results

### Pollen viability during flowering differed among jasmine petal-types

**Fig 2:**
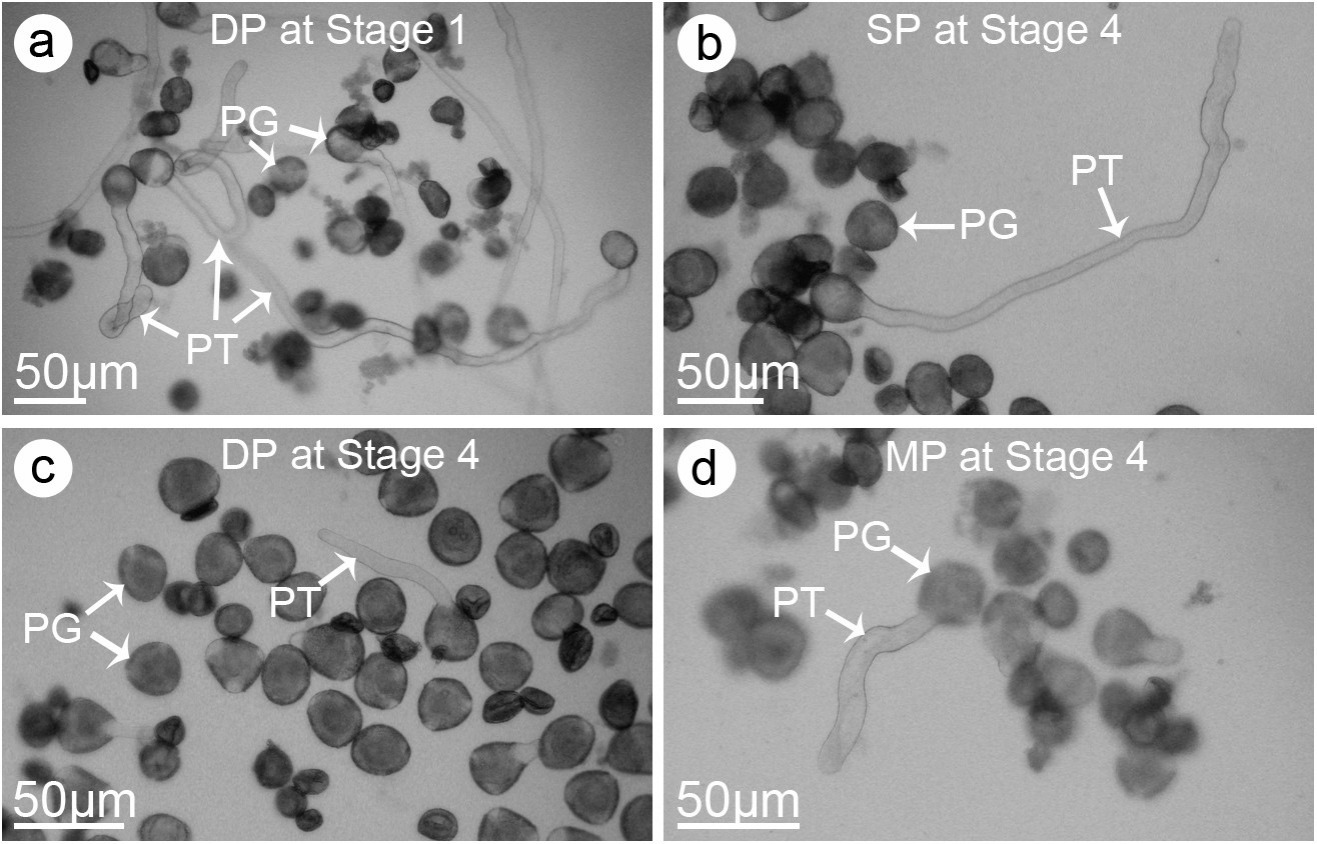
Pollen *in vitro* culture of three jasmine petal-types at different developmental stages. (**a**) Germination of the double-petal (DP) jasmine pollen grains in *Stage 1* (i.e., one day before flowers partially opened). (**b**) Germination of the single-petal (SP) jasmine pollen grains in *Stage 4* (i.e., one day post flowers fully opened). (**c**) Germination of the DP jasmine pollen grains in *Stage 4*. (**d**) Germination of the multi-petal (MP) jasmine pollen grains in *Stage 4*.

To assess whether the different jasmine phenotypes were associated with differences in pollen viability, pollen grains at four flower developmental stages were germinated *in vitro*. The highest pollen germination rates for the SP and DP jasmine samples (i.e., 10.1% and 20.3%, respectively) were detected during *Stage 1* (Fig 2A; Table 1). The germinability of the SP jasmine pollen decreased to 9.0% in *Stage 2*, 5.3% in *Stage 3*, and 3.2% in *Stage 4* (Fig 2B; Table 1). For the DP jasmine pollen, the germination rates decreased to 16.7%, 9.6%, and 5.6% in *Stages 2*, *3* and *4*, respectively (Fig 2C; Table 1). In contrast, the MP jasmine pollen germination rate was 4.1% in *Stage 1*. This was followed by an increase to 5.4% in *Stage 2*, and a decrease to 4.0% and 1.8% in *Stages 3* and *4*, respectively (Fig 2D; Table 1). A two-way ANOVA showed that the petal-type, developmental stage and their interaction had significant effects on pollen viability (*P* < 0.01). The pollen germinability varied among petal-types (rank order: DP > SP > MP) and flower developmental stages. Therefore, in the following pollination experiments, the SP and DP jasmine pollen grains were collected in *Stage 1*, whereas the MP jasmine pollen grains were harvested during *Stage 2*.

**Table 1:** Pollen viability of three jasmine petal-types during flowering.

Phenotype	Pollen viability (%)			
	Stage 1^a^	Stage 2	Stage 3	Stage 4
Single-petal type	10.1±1.4A^b^	9.0±0.6B	5.3±0.7C	3.2±0.5D
Double-petal type	20.3±1.9A	16.7±0.8B	9.6±1.2C	5.6±0.7D
Multi-petal type	4.1±0.7B	5.2±0.6A	4.0±0.3B	1.8±0.4C

^a^The definition of *Stages 1*, *2*, *3* and *4* was following: one day before flowers partially opened, flowers partially opened, flowers fully opened, and one day post flowers fully opened, respectively.

^b^Different capital letters represent significant differences at the level of P<0.01 between different developmental stages (mean ± SD).

### Stigma receptivity during flowering differed among jasmine petal-types

**Fig 3:**
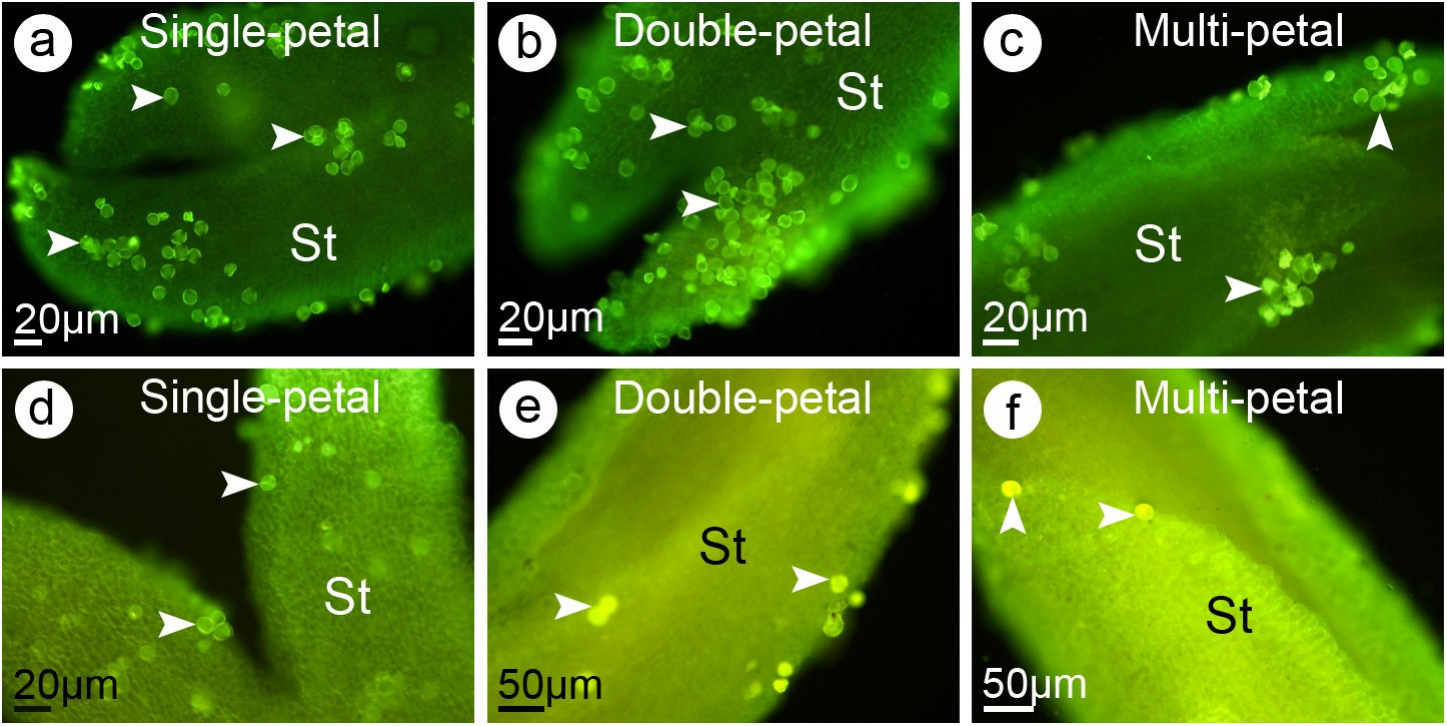
The fluorescent observation of pollen grains adhered on stigmas of jasmine three petal-types at different developmental stages. (**a**) The single petal (SP) jasmine pistil in *Stage 2* (i.e., partially opened flowers). The pollen grains were indicated by *arrow heads*. (**b**) The double petal (DP) jasmine pistil in *Stage 2*. The pollen grains were indicated by *arrow heads*. (**c**) The multi-petal (MP) jasmine pistil in *Stage 1* (i.e., one day before flower partially opened). The pollen grains were indicated by *arrow heads*. (**d**) The SP jasmine pistil in *Stage 4* (i.e., one day post flower fully opened). (**e**) The DP jasmine pistil of at *Stage 4*. The pollen grains were indicated by *arrow heads*. (**f**) The MP jasmine pistil in *Stage 4*. The pollen grains were indicated by *arrow heads*. Abbreviation: St, stigma

To determine whether the different jasmine phenotypes resulted in differences in stigma receptivity, the adhered and/or germinated pollen grains on each stigma were analyzed and compared. The two-way ANOVA showed that the flowering stage had a significant effect (*P*<0.01) on the percentage of pollen grains on each stigma, but the petal phenotype and the interaction between petal phenotype and flowering stage had no significant effects on the germination of pollen grains (*P*>0.05). In the SP and DP jasmine samples, the highest percentages (i.e., 23.8% and 21.4%, respectively) were recorded for the stigmas in *Stage 2* (Table 2). In contrast, the highest percentage for the MP jasmine pollen (i.e., 11.1%) was recorded for stigmas in *Stage 1* (Fig 3A–3C; Table 2). In fully opened flowers, the percentages of adhered and/or germinated pollen grains decreased to 2.8%, 2.5% and 1.8% for SP, DP and MP jasmine samples, respectively (Fig 3D–3F; Table 2). Therefore, in *Stages 1* to *3* (i.e., before and during anthesis), the pistils were fairly receptive to pollen grains, but the receptivity quickly decreased after anthesis. The most receptive SP and DP jasmine pistils were observed in *Stage 2*, while the most receptive MP jasmine pistils were detected in *Stage 1* (Table 2). Thus, the pistil receptivity varied among the petal-types (rank order: SP > DP > MP) and flower developmental stages. The *Stage 2* pistils of SP and DP jasmine samples, and the *Stage 1* pistils of the MP jasmine samples were selected as pollen receptors in the subsequent crosses to determine pollen-stigma interactions.

**Table 2:** Germination ratio of pollen grains on the stigmas at different flowering stages after artificial pollination of three jasmine petal-types.

Flowering stage	Ratio of germinated pollen grains on the stigmas (%)		
	SP × SP^b^	DP × DP	MP × MP
Stage 1^a^	10.6±2.2C^c^	6.9±1.5C	11.1±1.6A
Stage 2	23.8±3.5A	21.4±3.6A	8.1±1.1B
Stage 3	18.5±2.5B	13.9±1.8B	4.3±0.9C
Stage 4	2.8±0.9D	2.5±1.0D	1.8±0.5D

^a^The definition of flowering stages 1, 2, 3 and 4 was following: one day before flower partially opened, flower partially opened, flower fully opened, and one day post flower fully opened, respectively.

^b^SP, DP and MP represent the single-, double-, and multi-petal jasmine phenotypes, respectively.

^c^Different capital letters represent significant differences at the level of P<0.01 between different flowering stages (mean ± SD).

### Different crossing combinations exhibited distinct pollen-stigma compatibilities

**Fig 4:**
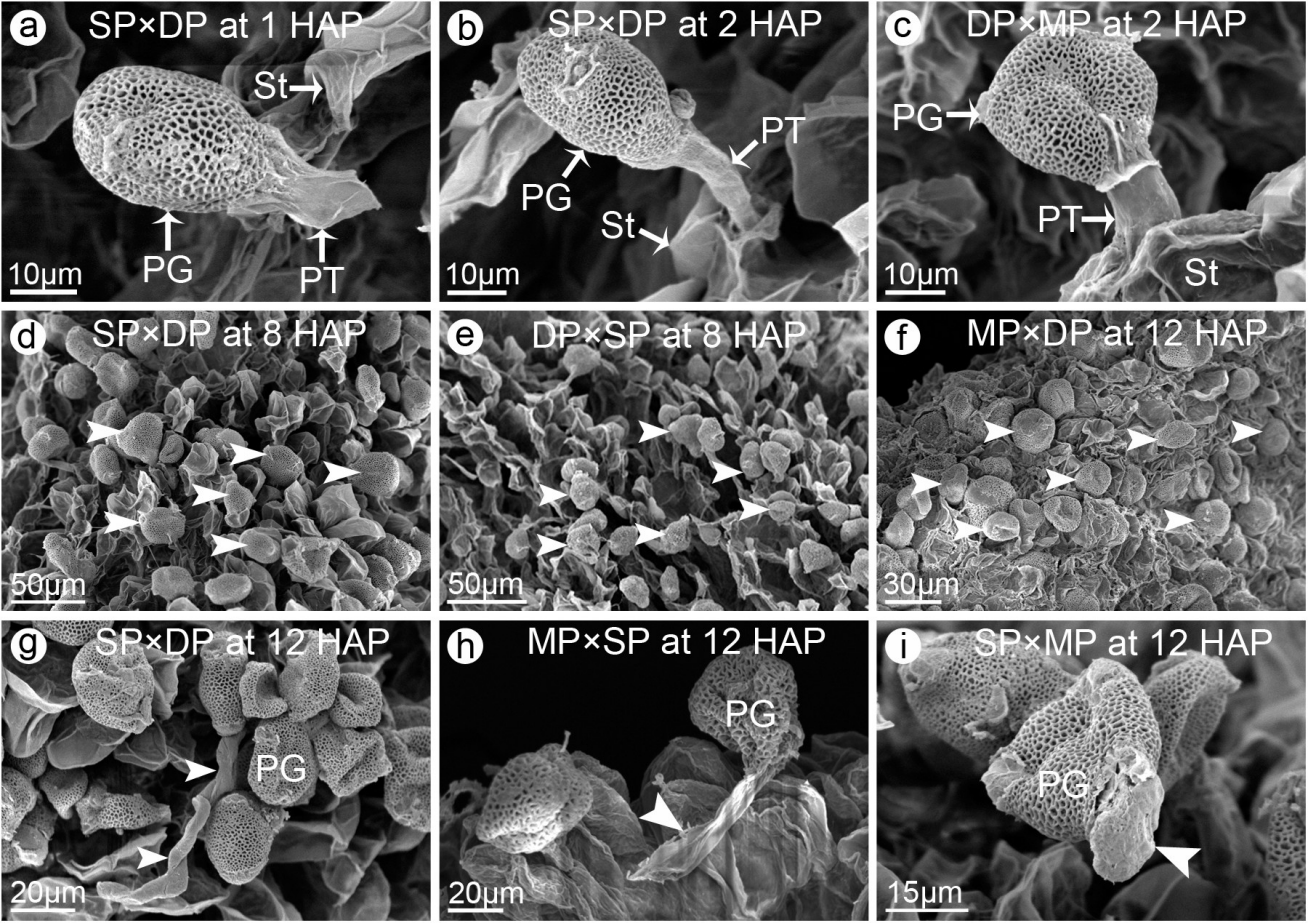
The SEM observation of pollen germination and pollen tube growth in different cross combinations among single-petal (SP), double-petal (DP) and multi-petal (MP) jasmine plants. (**a**) At 1 h after pollination (HAP), in SP × DP (pollen receptor × pollen donor), the pollen tube had just emerged from the germinal aperture. (**b**) At 2 HAP, in SP × DP, the pollen tube had penetrated into the stigma. (**c**) At 2 HAP, in DP × MP, the pollen tube had penetrated into the stigma. (**d**) At 8 HAP, in SP × DP, the number of geminated pollen grains (*arrow heads*) reached the peak. (**e**) At 8 HAP, in DP × SP, the number of geminated pollen grains (*arrow heads*) reached the peak. (**f**) In MP × DP, the number of geminated pollen grains (*arrow heads*) reached the peak at 12 HAP. (**g**) In SP × DP, a circulated and twisted pollen tube on the stigmatic surface at 12 HAP. (**h**) In MP × SP, a collapsed pollen tube on the stigma at 12 HAP. (**i**) In SP × MP, a pollen tube enlarged abnormally just after emerging from the aperture at 12 HAP. Abbreviations: PG, pollen grain; PT, pollen tube; St, stigma

**Fig 5:**
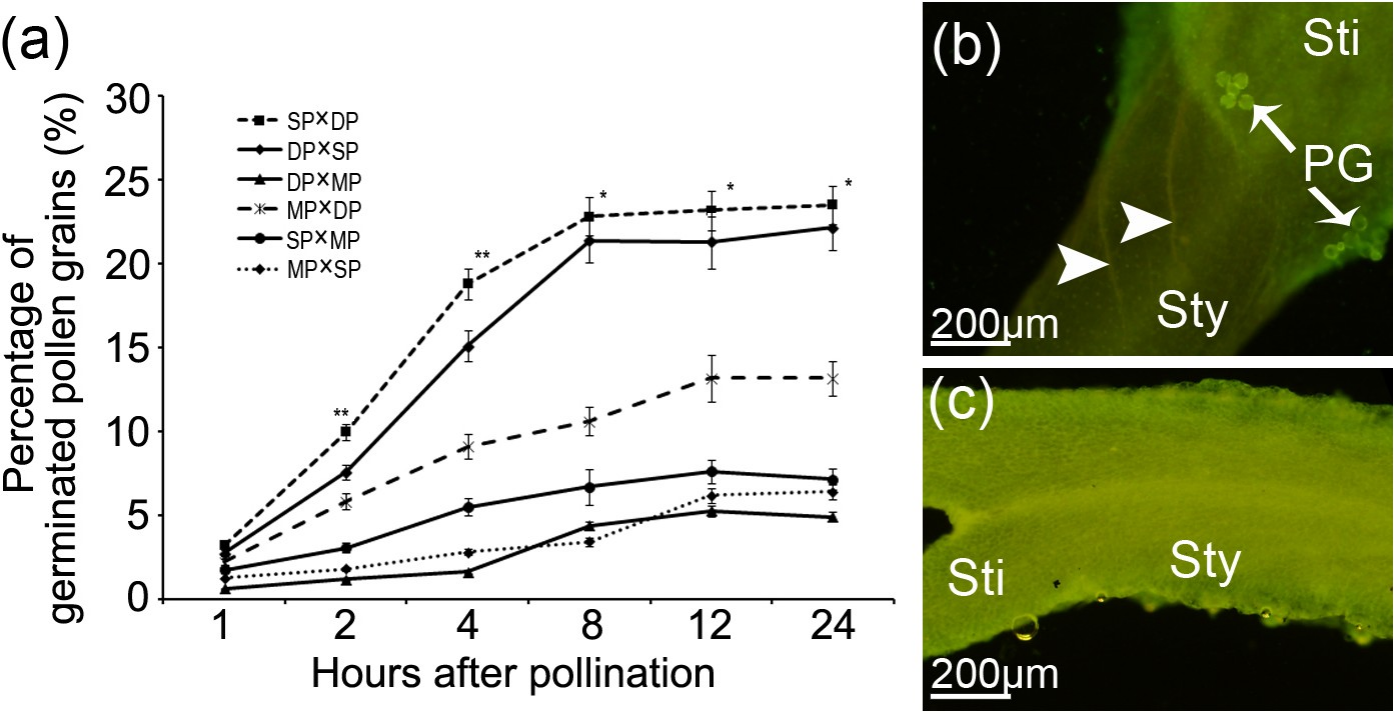
The fluorescent microscope observation of pollen germination and pollen tube growth in different cross combinations among single-petal (SP), double-petal (DP) and multi-petal (MP) jasmine plants. (**a**) The percentage of pollen grains on stigmas at different hours after pollination (HAP). ** and * represent significant differences at the level of P < 0.01 and 0.05, respectively. (**b**) A style of SP jasmine crossed with DP jasmine exhibiting several pollen tubes (*arrowheads*) in the upper position of the style at 24 HAP. (**c**) A style of DP jasmine crossed with MP jasmine exhibiting no pollen tube in the style at 24 HAP. Abbreviations: PG, pollen grain; Sti, stigma; Sty, style.

We analyzed pollen germination and pollen tube growth in six reciprocal crosses to compare the pollen-stigma compatibilities among jasmine cultivars with different petal-types. For all combinations, most of the pollen grains did not germinate within the first hour following pollination, and the pollen tubes had just emerged from the germinal apertures in only a few pollen grains (Fig 4A). However, by 2 HAP, some pollen tubes had penetrated into the stigma (Fig 4B and 4C). In the SP × DP and DP × SP reciprocal crosses, the percentages of pollen grains on the stigmas increased quickly during the first 8 HAP. Thereafter, the percentages increased slightly until 24 HAP (Fig 4D and 4E; Fig 5A). However, in the other four cross combinations (i.e., DP × MP, MP × DP, SP × MP, and MP × SP), the percentage of pollen grains on each stigma increased slowly until 12 HAP (Fig 4F), before leveling off slightly (Fig 5A). This suggested the pollen grains and stigmas were relatively incompatible. Meanwhile, significant differences in the germinated pollen grains were observed among the different crosses. For example, at 8 HAP, 22.8% and 21.4% of the pollen grains were present on the stigmas for the SP × DP and DP × SP crosses. In contrast, only 4.4%, 10.6%, 6.7%, and 3.6% of the pollen grains were detected on the stigmas of the DP × MP, MP × DP, SP × MP, and MP × SP crosses, respectively (Fig 5A). In these four cross combinations, the percentage of pollen grains that had germinated at 12 HAP was only 5.3%, 13.2%, 7.6% and 6.2%, respectively, which was considerably lower than the corresponding germination rate of the SP × DP (23.2%) and DP × SP (21.3%) crosses (Fig 5A). During pollen germination and pollen tube growth, some abnormal phenomena were observed in different cross combinations. For example, a few pollen tubes appeared twisted or coiled on the surface of stigmas (Fig 4G). In some instances, instead of penetrating into a stigma, the tube collapsed and went flat (Fig 4H). Occasionally, the newly emerged pollen tube enlarged abnormally and then stopped growing (Fig 4I). The SEM results suggested that different cross combinations produced different pollen-stigma compatibilities (rank order: SP × DP > DP × SP > MP × DP > SP × MP > MP × SP > DP × MP) (Fig 5A). Although the SEM images revealed that some pollen tubes could penetrate the stigmatic surface at 12 HAP, only a few pollen tubes could be observed in the upper parts of the style at 24 HAP (Fig 5B). In most cases, pollen tubes were not detected in the stylar-transmitting tissues until 24 HAP (Fig 5C).

### Reciprocal crosses resulted in unfertilized embryo sacs following pollination

**Fig 6:**
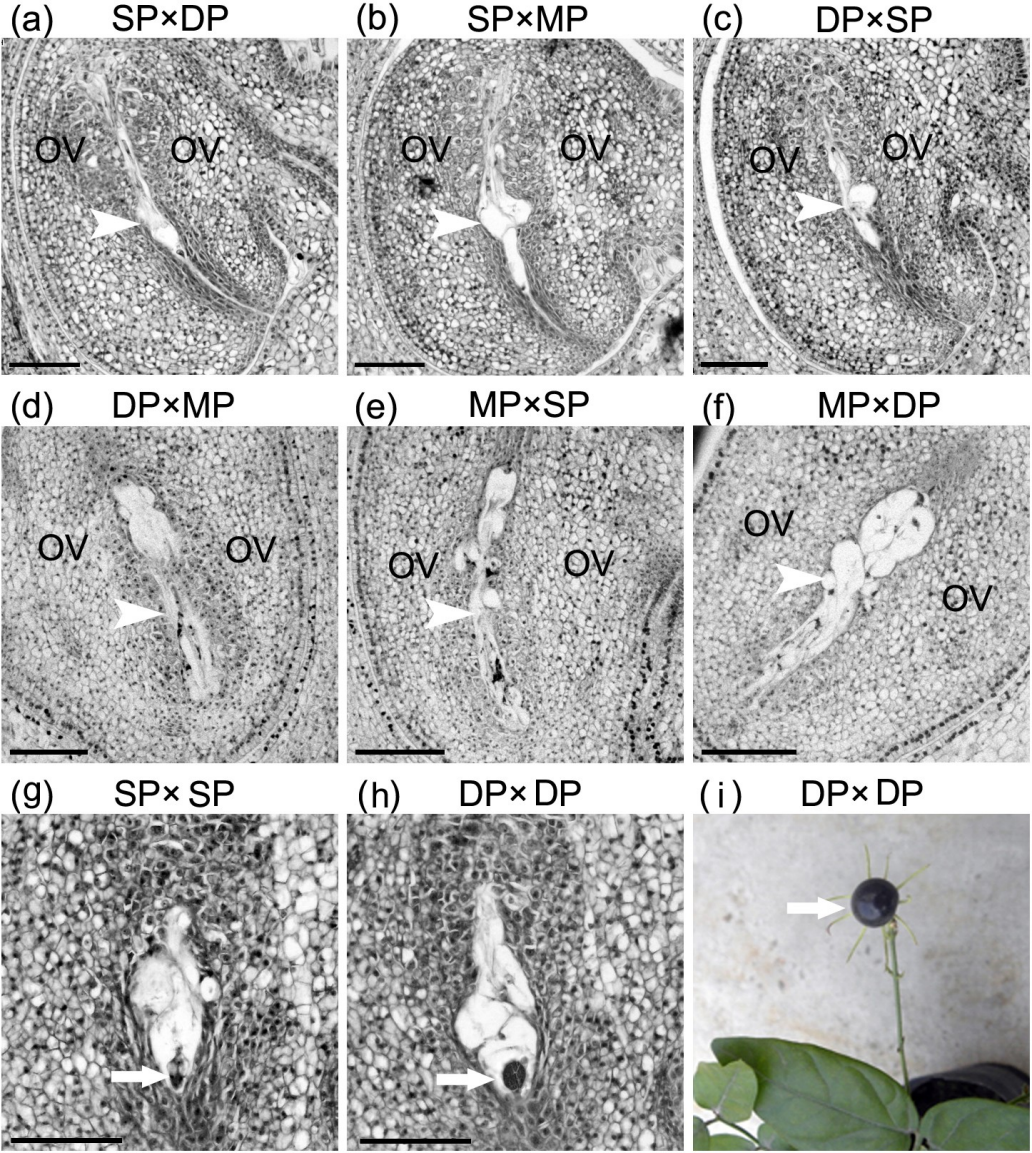
Development of the pollinated embryo sacs in reciprocal crosses among single-petal (SP), double-petal (DP) and multi-petal (MP) jasmine plants and self-pollinated plants. (**a**) Unfertilized embryo sac (*arrow head*) of SP × DP at 3 d after pollination (DAP). (**b**) Unfertilized embryo sac (*arrow head*) of SP × MP degenerated at 5 DAP. (**c**) Unfertilized embryo sac (*arrow head*) of DP × SP at 3 DAP. (**d**) Unfertilized embryo sac (*arrow head*) of DP × MP degenerated at 5 DAP. (**e**) Unfertilized embryo sac (*arrow head*) of MP × SP at 3 DAP. (**f**) Unfertilized embryo sac (*arrow head*) of MP × DP degenerated at 4 DAP. (**g**) A multi-cellular embryo (*arrow*) of SP × SP at 5 DAP. (**h**) A globular embryo (*arrow*) of DP × DP at 10 DAP. (**i**) A mature fruit (*arrow*) of DP × DP. Abbreviation: OV: ovule. Scale bars: 100 μm

Anatomic observations of the ovules of six cross combinations revealed that none of their embryo sacs were fertilized by 3 DAP (Fig 6A, 6C and 6E). Furthermore, the unfertilized embryo sacs in the SP, DP and MP jasmine plants began to degenerate at 5, 5 and 4 DAPs, respectively (Fig 6B, 6D and 6F). Simultaneously, no pollen tubes were observed in the ovules at the same time points (Fig 6A–6F). However, some self-pollinated embryo sacs would develop normally. The multi-cellular embryo could be observed in the self-crossed SP ovules at 5 DAP (Fig 6G). In self-pollinated DP plants, the embryos would develop to a globular stage by 10 DAP (Fig 6H), and some of them formed mature fruits (Fig 6I).

### Pistil cells of the three jasmine petal-types exhibited different ultrastructural changes during flowering

**Fig 7:**
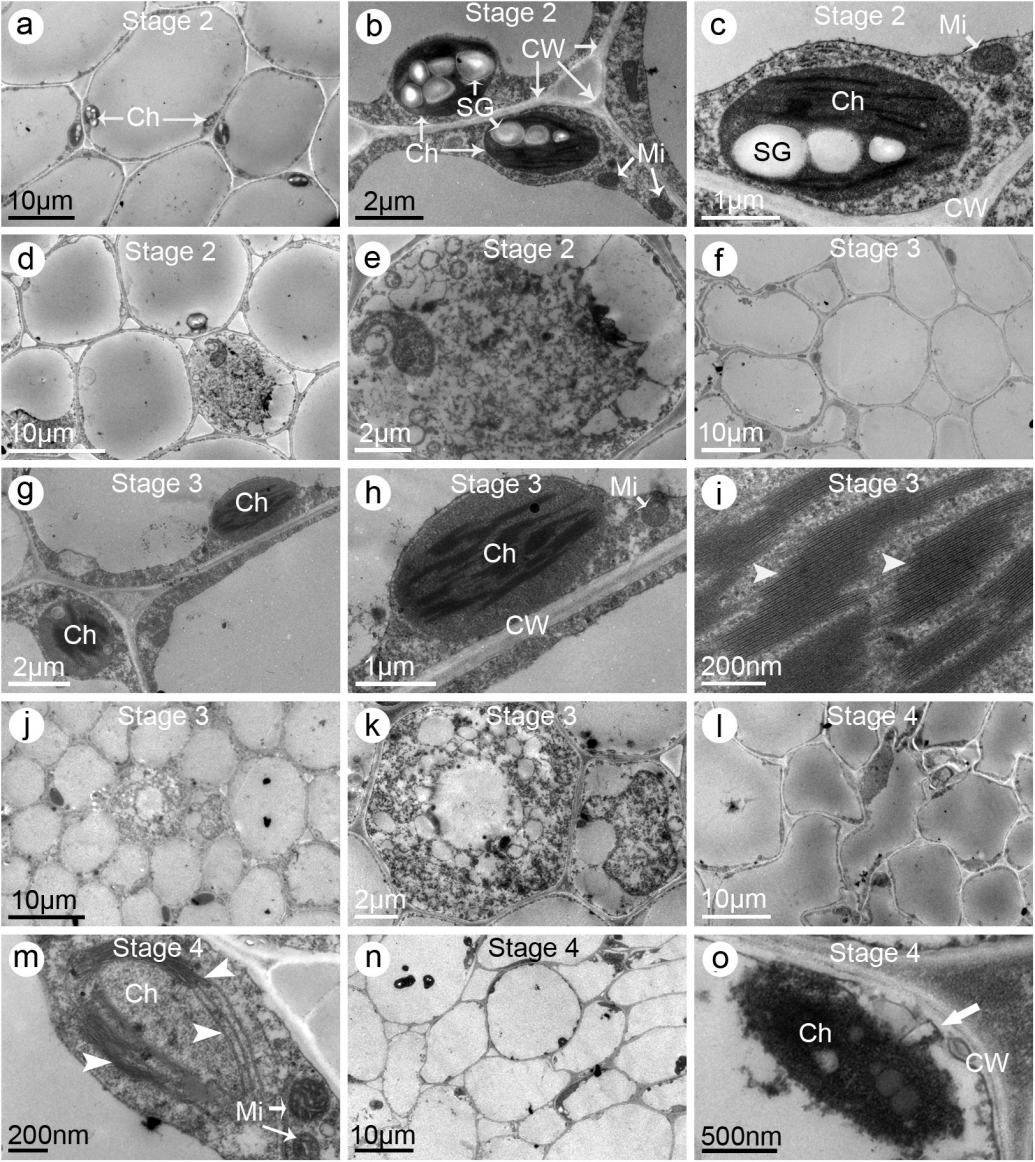
Electron micrographs of stigmas and styles of the single-petal (SP) jasmine plants. (**a–c**) Stigmas sampled from the partially opened flowers (i.e., *Stage 2*). (**a**) Multi-cellular section exhibiting the ultrastructural characteristics. Note the starch granules in the chloroplasts. (**b**) Enlarged graph of (**a**) showing the organelles including chloroplasts, starch granules, and mitochondria. (**c**) A single chloroplast. (**d–e**) Styles sampled at *Stage 2*. (**d**) Multi-cellular section exhibiting the ultrastructural characteristics. (**e**) Enlarged graph of (**d**) showing the transfer cell. (**f–i**) Stigmas sampled from the fully opened flowers (i.e., *Stage 3*). (**f**) Multi-cellular section exhibiting the ultrastructural characteristics. Notice fewer starch granules were observed. (**g**) Enlarged graph of (**f**) showing several cells and their organelles. (**h**) Enlargement of (**g**) showing a single chloroplast. (**i**) Enlargement of (**h**) showing the grana and lamellae (*arrow heads*). (**j–k**) Styles sampled at *Stage 3*. (**j**) Multi-cellular section exhibiting the ultrastructural characteristics. (**k**) Enlarged graph of (**j**) showing the transfer cell. (**l–m**) Stigmas sampled from the flowers at one day post fully opened (i.e., *Stage 4*). (**l**) Multi-cellular section exhibiting its ultrastructural characteristics. Notice the cells were of irregular shape and intercellular spaces were larger than normal. (**m**) A single chloroplast showing fewer grana and lamellae (*arrow heads*) than normal. (**n–o**) Styles sampled at *Stage 4*. (**n**) Multi-cellular section exhibiting the ultrastructural characteristics. (**o**) A single chloroplast showing the degenerating grana and lamellae. Notice the plasmolysis (*arrow*) occurred. Abbreviations: Ch, chloroplast; CW, cell wall; Mi, mitochondria; SG, starch granule

Ultrastructural observations revealed that the pistil cells underwent obvious changes during the development of flowers. Until the end of *Stage 2*, the stigmatic cells of SP jasmine plants were involved in metabolic activities, as indicated by the presence of complete organelles, including chloroplasts and mitochondria, and numerous starch granules (Fig 7A–7C). The stylar cells also contained dense components similar to the stigmatic cells (Fig 7D and 7E). However, starch granules were not observed in the stigmatic cells of *Stage 3* flowers, while the grana and lamellae maintained their regular shapes (Fig 7F–7I). The stylar cells exhibited similar changes, with an almost complete lack of starch granules (Fig 7J). Additionally, the cells exhibited obvious signs of degeneration, such as the development of different sized cavities (Fig 7K). The stigma cells started to deform and senesce during *Stage 4*, and the chloroplasts contained fewer grana and lamellae than normal (Fig 7L and 7M). In stylar cells, the organelles degenerated and plasmolysis was observed (Fig 7N and 7O).

**Fig 8:**
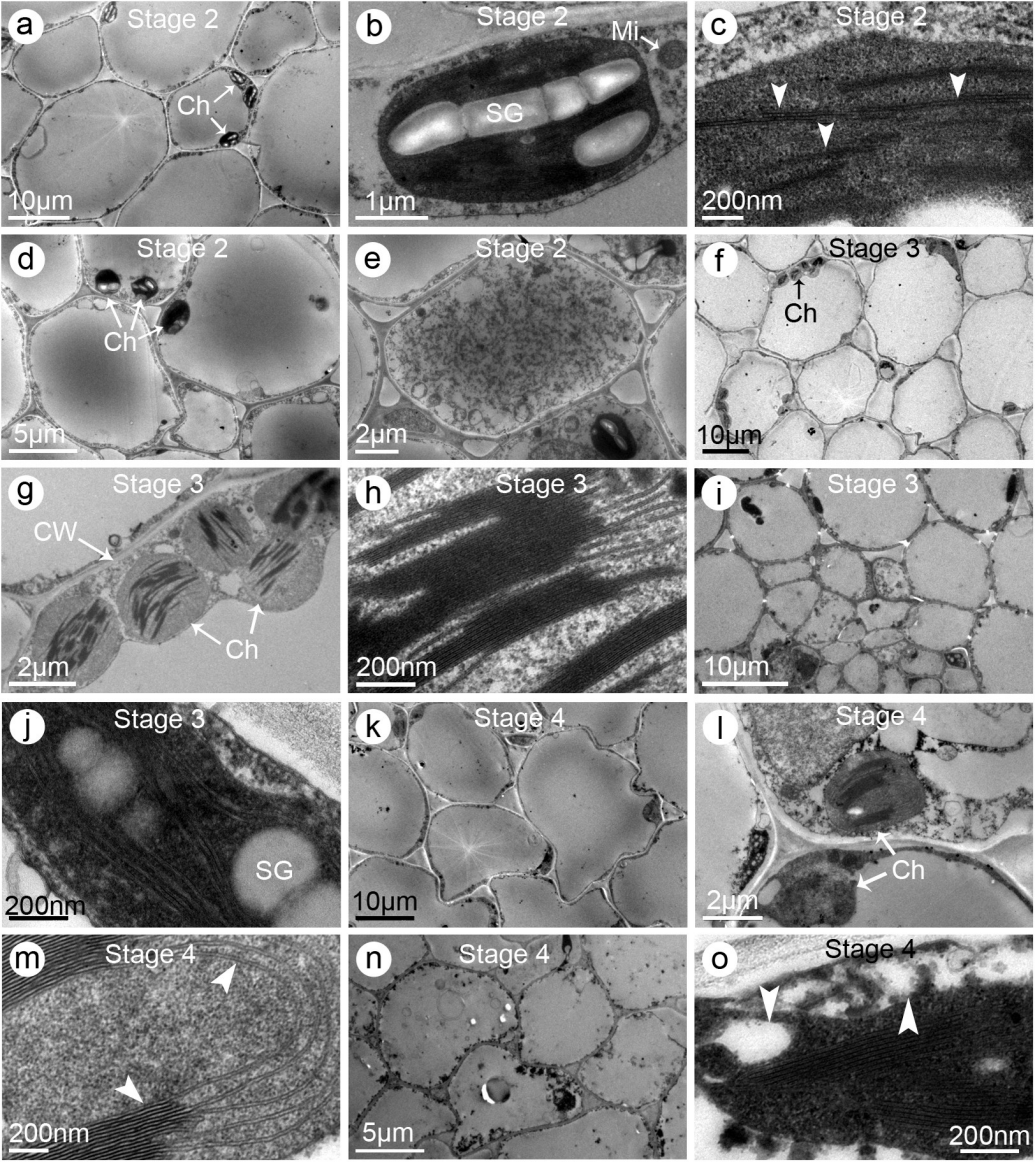
Electron micrographs of stigmas and styles of the double-petal (DP) jasmine plants. (**a–c**) Stigmas sampled from partially opened flowers (i.e., *Stage 2*). (**a**) Multi-cellular section exhibiting the ultrastructural characteristics. Several starch granules were observed in each chloroplast. (**b**) Enlarged graph of (**a**) showing a single chloroplast with starch granules. (**c**) Enlargement of the chloroplast showing the grana and lamellae (*arrow heads*). (**d–e**) Styles sampled at *Stage 2*. (**d**) Multi-cellular section exhibiting the ultrastructural characteristics. Note several starch granules in each chloroplast. (**e**) The transfer cell. (**f–h**) Stigmas sampled from fully opened flowers (i.e., *Stage 3*). (**f**) Multi-cellular section exhibiting the ultrastructural characteristics. Note starch granules were not observed although chloroplasts still existed. (**g**) Enlarged graph of (**f**) showing several chloroplasts. (**h**) Enlargement of a chloroplast showing the grana and lamellae. (**i–j**) Styles sampled at *Stage 3*. (**i**) Multi-cellular section exhibiting the ultrastructural characteristics. (**j**) Enlarged graph of (**i**) showing a chloroplast. Notice the grana and lamellae were empty and fewer than normal. (**k–m**) Stigmas sampled from flowers at one day post fully opened (i.e., *Stage 4*). (**k**) Multi-cellular section exhibiting the ultrastructural characteristics. Notice the shape of cells was irregular. (**l**) Enlarged graph of (**k**) showing the cellular organelles. (**m**) A single chloroplast with flaccid grana and lamellae (*arrow heads*). (**n–o**) Styles sampled at *Stage 4*. (**n**) Multi-cellular section exhibiting the ultrastructural characteristics. Notice the cells had degenerated, and the organelles were fuzzy and unclear. (**o**) A degraded chloroplast. Notice the plasmolysis (*arrow heads*) occurred and sized cavities had formed. Abbreviations: Ch, chloroplast; CW, cell wall; Mi, mitochondria; SG, starch granule

The pistil cell changes in the DP jasmine plants were similar to those of the SP plants. In the *Stage 2* stigmas, several starch granules were observed in each chloroplast, and the grana and lamellae were clear and complete (Fig 8A–8C). Meanwhile, starch granules were detected in the stylar cells (Fig 8D), which also contained a dense cytoplasm with abundant organelles (Fig 8E). However, by *Stage 3*, starch granules were not clearly observed in the stigmatic cells, although chloroplasts were still present, with normal-shaped grana and lamellae (Fig 8F–8H). The stylar cells were obviously degenerating, with several large intercellular spaces forming (Fig 8I). Additionally, starch granules were observed in only a few chloroplasts (Fig 8J). By *Stage 4*, the stigmatic cells contained only a few chloroplasts. Most of the organelles had degenerated, and the grana and lamellae were flaccid (Fig 8K–8M). The stylar cells degenerated so severely that the cellular organelles could not be clearly identified (Fig 8N). Furthermore, plasmolysis had occurred, and different sized cellular cavities had formed (Fig 8O).

**Fig 9:**
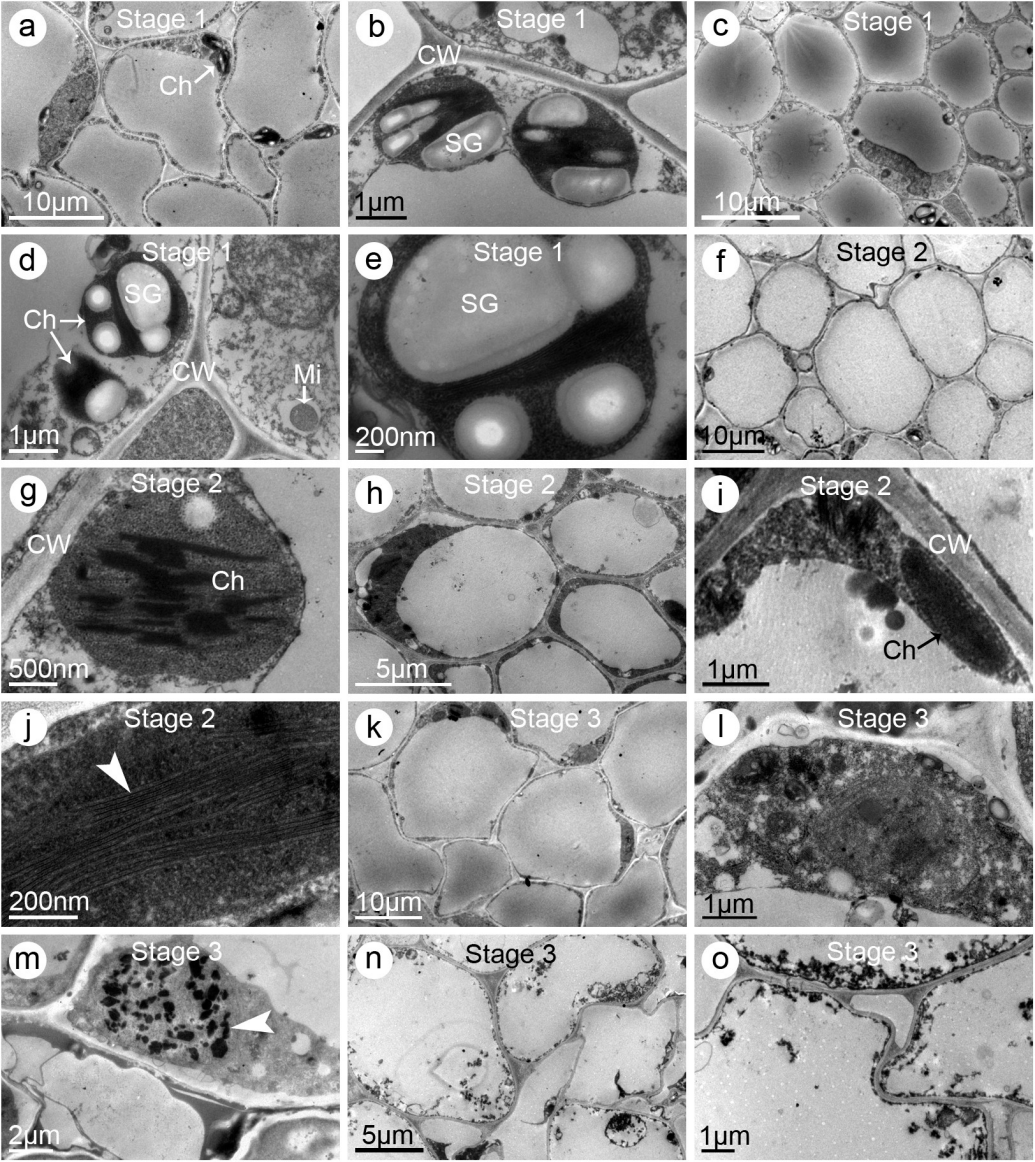
Electron micrographs of stigmas and styles of the multi-petal (MP) jasmine plants. (**a–b**) Stigmas sampled from the flowers at one day before partially opened (i.e., *Stage 1*). (**a**) Multi-cellular section exhibiting the ultrastructural characteristics. Note many starch granules were observed. (**b**) Enlarged graph of (**a**) showing the chloroplasts and starch granules. (**c–e**) Styles sampled at *Stage 1*. (**c**) Multi-cellular section exhibiting the ultrastructural characteristics. Notice the cells contained dense cytoplasm. (**d**) Enlargement of the cell showing its organelles. (**e**) Enlargement of a single chloroplast with starch granules. (**f–g**) Stigmas sampled from the partially opened flowers (i.e., *Stage 2*). (**f**) Multi-cellular section exhibiting the ultrastructural characteristics. Note the chloroplasts were fewer than normal and starch granules were nearly disappeared. (**g**) Enlarged graph of a single chloroplast with irregular grana and lamellae. (**h–j**) Styles sampled at *Stage 2*. (**h**) Multi-cellular section exhibiting the ultrastructural characteristics. (**i**) Enlarged graph of (**h**) showing the cellular containers. (**j**) Enlarged graph of (**h**) showing the lamellae (*arrow heads*). (**k–m**) Stigmas sampled from the fully opened flowers (i.e., *Stage 3*). (**k**) Multi-cellular section exhibiting its ultrastructural characteristics. Notice no starch granules were observed in the cells. (**l**) Enlarged graph of (**k**) showing the degenerated cellular organelles. (**m**) Another cell showing the degraded nucleus and its apoptotic bodies (*arrow heads*). (**n–o**) Styles sampled at *Stage 3*. (**n**) Multi-cellular section exhibiting its ultrastructural characteristics. Notice the cellular organelles had entirely degenerated. (**o**) Enlargement of the stylar cell showing its degenerated containers. Abbreviations: Ch, chloroplast; CW, cell wall; Mi, mitochondria; SG, starch granule

The ultrastructural characteristics of the pistil cells of MP jasmine plants differed from those of SP and DP plants. Large starch granules were observed in the stigmatic and stylar cells during *Stage 1* (Fig 9A–9E). However, by *Stage 2*, only a few small starch granules were detected in the stigmatic cells, while the stylar cells completely lacked any starch granules (Fig 9F–9H). Although whole chloroplasts maintained their regular shape, the grana and lamellae were already flaccid (Fig 9I and 9J). Subsequently, in the *Stage 3* stigmatic cells, the chloroplasts were degenerated, and starch granules were absent (Fig 9K and 9L). Additionally, nuclei were also degraded, and only small apoptotic bodies were observed (Fig 9M). The stylar cells severely degenerated, and almost all of the organelles had degraded (Fig 9N and 9O).

## Discussion

### Pre-fertilization reproductive barriers affect jasmine crosses

Reproductive barriers are generally subdivided into pre- and post-fertilization barriers (also called as pre- and post-zygotic barriers) depending on whether fertilization occurs normally [24]. These two kinds of barriers are respectively characterized by incompatibility (incongruity) and embryo abortion (hybrid breakdown) [19,24]. Pre-fertilization barriers are usually based on the fertility of the parents and pollen-pistil interactions (including pollen germination, pollen tube growth, and the callose reaction on stigma), which are useful indicators of compatibility and seed set [18,25]. For example, the relatively lack of germinated pollen grains, the presence of twisted and coiled pollen tubes, and the considerable deposition of callose on the stigmatic surface were considered to represent pre-fertilization barriers because the pollen grains were incompatible with the pistils in these cases [18,20,25]. In the present study, although some pollen grains germinated normally on the maternal stigmas, they were arrested in the stigmas or styles, and no pollen tubes reached the ovaries. Furthermore, the embryo sacs of all female parents remained unfertilized prior to degenerating. Therefore, the crosses between these jasmine petal-types were affected by pre-fertilization reproductive barriers.

### Low or easily lost pollen fertility is one of the pre-fertilization barriers affecting hybrid set

Pollen viability is considered an important pre-fertilization factor influencing seed or fruit production [26]. Low pollen fertility can decrease the germination rate on stigmas. Consequently, when pollen grains with poor viability pollinate a stigma, the probability of fertilization is usually low, resulting in decreased seed production [10,27]. Our results indicate that the pollen grains in the three jasmine petal-types exhibited poor germinability, with the highest and lowest percentages being 20.3% (i.e., DP jasmine in *Stage 1*) and 1.8% (i.e., MP jasmine in *Stage 4*), respectively (Table 1). Additionally, pollen fertility is unstable during flowering and is easily lost at anthesis. Our results are consistent with those of previous studies that revealed the poor viability of jasmine pollen grains [12–14], and they also confirmed that poor viability is a pre-fertilization barrier affecting hybrid production.

Pollen grains that easily become non-viable have been reported for many plants. For example, lotus pollen grains remain viable only for several hours after anther dehiscence [28]. We observed that the three jasmine petal-types exhibited obvious differences in pollen viability. The pollen grains from DP jasmine plants were the most viable, while the MP jasmine pollen grains were the least viable (Table 1). Therefore, jasmine pollen viability varies depending on the flower developmental stage and petal type. Based on pollen fertility, DP jasmine plants may be more suitable as the male parent than the other two jasmine cultivars. Nevertheless, jasmine pollen viability is low and easily lost. Our data reveal that selecting a suitable time to collect fertile pollen grains for pollination is critical for overcoming the pre-fertilization barriers in jasmine crosses.

### Early senescence of pistil cells and low pistil receptivity are important pre-fertilization barriers affecting hybrid set

Pistil receptivity is an important determinant of successful pollination and fertilization in seed plants. Poor pistil receptivity is one of the main factors responsible for low seed output in many crosses, including those involving chrysanthemum, sorghum, and lotus [9,15,29]. Our results revealed significant differences in pistil receptivity among the three jasmine petal-types and the four developmental stages. Furthermore, we determined that stigmas in fully opened flowers are almost unreceptive to pollen. Therefore, poor pistil receptivity is another important pre-fertilization barrier that inhibits hybrid set in jasmine crosses. Thus, determining when jasmine pistils are the most receptive to pollen is important for mitigating the pre-fertilization barriers. According to pistil receptivity, SP jasmine plants seem to be more suitable as the female parent than the DP or MP jasmine plants.

Previous studies on other plant species concluded that pistil cell ultrastructural changes during flowering influences pistil receptivity [23,30]. Our ultrastructural observations provide new insights into the changes in pistil receptivity. Under ideal conditions for stigma receptivity, the stigmatic and stylar cells were highly metabolically active, and produced large starch granules, intact chloroplasts, and clear grana lamellae. Along with significant decreases in stigma receptivity after flowers bloomed, the stigmatic and stylar cells underwent death. Anatomical observations of the pollinated ovaries indicated that the embryo sacs were unfertilized and pollen tubes were unable to reach the ovules (Fig 6). These results indicate that the pollen tubes were arrested or grew very slowly in the pistils after they entered the stigma. Undoubtedly, early and rapid senescence of pistils is harmful for pollen adhesion and germination as well as pollen tube growth. Considering only one pistil and numerous pollen grains are developed simultaneously in one flower, pistil receptivity is more important than pollen viability in jasmine crosses. Pistil ultrastructural features differed depending on petal types and flower developmental stages. Therefore, clarifying the dynamic ultrastructural changes occurring during pistil development may be useful for characterizing the variability in receptivity and determining the most appropriate stage for manual pollination. Our findings not only revealed the cytological mechanism underlying poor pistil receptivity, they also confirmed the importance of pistils for the pre-fertilization reproductive barriers in jasmine crosses.

### Pollen-stigma compatibility among jasmine petal-types varied with the parents

In the present study, we analyzed the pollen-stigma interactions among three jasmine petal-types. We observed that many pollen grains germinated normally, and most of the pollen tubes penetrated into the stigmas within 24 HAP. Thus, the pollen grains and stigmas can be considered compatible (or at least partly compatible). However, the germination rate varied significantly, and depended on the parents. Specifically, the germination rate was influenced by the petal phenotypes of the parents. The compatibility between pollen grains and stigmas was higher for the SP and DP cross than for the other parental combinations (i.e., SP and MP, and DP and MP). Additionally, we observed that the compatibility varied with the cross order of parents. For example, the compatibility of SP × DP was higher than that of DP × SP (Fig 5A). Therefore, selecting suitable parents for jasmine hybridizations may help mitigate the pre-fertilization barriers.

Reciprocal crosses exhibiting different compatibilities have ever been reported for many plant species. For example, *Zinnia angustifolia* crossed with *Z*. *elegans* was compatible, but *Z*. *elegans* was not compatible with *Z*. *angustifolia* [31]. Similarly, the intergeneric hybridization between *Lolium perenne* and *Festuca arundinacea* was compatible, yet *F*. *arundinacea* crossed with *L*. *perenne* was incompatible [32]. Additionally, the seed setting rates were highly variable in the reciprocal cross between *Chrysanthemum morifolium* and *Ajania pacifica* because of differences in compatibilities [33]. The present study revealed that SP jasmine plants were better suited as the female parent than as the male parent, whereas DP jasmine plants exhibited the opposite pattern. Based on the compatibilities, the MP jasmine plants were not suitable as either the female or male parent. When MP jasmine plants were used as the female parent, DP jasmine plants were more appropriate as the male parent than the SP jasmine plants. This may be because of the greater viability of the DP jasmine pollen grains. In contrast, when MP jasmine plants served as the male parent in crosses with SP or DP jasmine plants, the compatibilities were low because of its poor pollen viability. Therefore, MP jasmine plants were always the least compatible parent for jasmine hybridizations. The SP jasmine pistils were more receptive to pollen than the DP jasmine pistils. Thus, the compatibility between SP and MP plants was higher than that between DP and MP. These observations may be useful for ensuring the most appropriate parents are selected for jasmine hybridization experiments.

Pre-fertilization barriers would be resulted by many factors including the parents’ reproductive organs and their interactions. Besides pollen viability, pistil receptivity and pollen-stigma compatibility, ovule sterility is also an important reason leading to low seed set in many flowering plants [19–20]. The previous study has shown that the development of ovules in DP jasmine plants was often abnormal [14]. Undoubtedly, the abnormal ovules were adverse to set hybrids. Therefore, the fertility of ovules in three petal-types of jasmine is valuable to investigate thoroughly in the future for an overall understanding of the pre-fertilization barriers in jasmine hybridizations.

### Phylogenetic relationships among different jasmine petal-types

Despite the horticultural significance of floral species that produce two or more petals, the mechanisms regulating the development of supernumerary petals have not been fully characterized. Thus, comparative studies have been conducted using plant species exhibiting different petal phenotypes to evaluate their phylogenetic relationships or clarify their origins [34,35]. Hybridizations are a means to generate new plant materials, but they are also potentially useful for determining the phylogenetic relationship between the parents to some extent. For example, when a chrysanthemum cultivar was crossed with *Ajania*, *Artemisia*, *Tanacetum*, *Leucanthemella*, or *Nipponanthemum* species, intergeneric hybrids were not easily produced except for the cross between chrysanthemum and the *Ajania* species [20,21,33,36]. It was difficult to generate hybrids between the *Chrysanthemum* species and *Artemisia*, *Nipponanthemum*, or *Leucanthemella* species, even when using an embryo rescue method [21,36]. These observations suggest that among these genera, *Chrysanthemum* is most closely related to *Ajania*. This relationship is supported by traditional morphological taxonomy as well as by molecular evidence [37,38]. Therefore, regarding pollen-stigma compatibility, it is likely that SP and DP jasmine are relatively closely related, while MP jasmine is more distantly related. A comparison of floral ontogeny in normal and double-flowered phenotypes of *Syringa vulgaris* suggested that the extra petal whorl in double-flowered plants was formed during evolution [35]. Therefore, it can be preliminarily speculated that the SP type may be the initially originated phenotype and the DP and MP phenotypes were formed from the SP plants. However, more detailed studies should be taken to elucidate the evolutionary relationships among the three phenotypes of jasmine. Nevertheless, this may be useful information for future research on jasmine genetics and the evolutionary developmental biology related to flowers.

## Conclusions

Pollen viability and pistil receptivity are variable and can be easily lost during flowering. Thus, they are associated with pre-fertilization reproductive barriers and differences in pollen-stigma compatibility in jasmine hybridizations. The early and rapid senescence of pistils plays a primary role in the incompatibility between pollen grains and stigmas. Our results presented herein provide new details regarding the reproductive biology relevant to the three jasmine petal-types, and may help clarify the reproductive barriers that exist during hybridizations. This information not only enables the development of effective strategies to overcome reproductive barriers (e.g., selecting suitable developmental stages and crossing order of parents and cutting pistils for pollination), it may also be useful for determining the phylogenetic relationships among jasmine varieties.
